# Bilateral carotid body paraganglioma: case report

**DOI:** 10.1590/S1516-31802000000100004

**Published:** 2000-01-02

**Authors:** Erasmo Simão da Silva, Fábio Lambertini Tozzi, Flávia Helena Matta de Paiva, Graziela de Almeida Sukys

**Keywords:** Carotid body, Paraganglioma, Cranial nerves, Corpo Carotídeo, Paraganglioma, Nervos cranianos

## Abstract

**CONTEXT:**

Surgical treatment of carotid body paragangliomas is a challenge to the surgeon because of the large vascularization of the tumor, involvement of the carotid vessels and the close anatomical relationship with the cranial nerves.

**CASE REPORT:**

A 63-year-old patient was submitted to resection of two carotid body paraganglioma tumors found in the right-side and left-side carotid bodies at the bifurcation of the common carotid arteries. Two surgeries were performed at different times and neither of them presented any morbidity. Arteriography was fundamental for diagnosis of the small, asymptomatic tumor on the right side.

**DESIGN:**

Case Report

## resumo

**CONTEXTO:**

O tratamento cirúrgico dos paragangliomas de corpo carotídeo é um desafio para o cirurgião devido à ampla vascularização do tumor, o envolvimento com os vasos carotídeos e a íntima relação anatômica com os nervos cranianos.

**RELATO DE CASO:**

Um paciente de 63 anos foi submetido à ressecção de dois paragangliomas, localizados nos corpos carotídeos do lado direito e esquerdo, junto às bifurcações das artérias carótidas comuns. As cirurgias foram em tempos diferentes e ambas sem morbidade. Para o diagnóstico do tumor pequeno do lado direito (assintomático) foi fundamental a arteriografia diagnóstica.

## INTRODUCTION

Surgical treatment of carotid body paragangliomas is a challenge to the surgeon because of the large vascularization of the tumor, involvement of the carotid vessels and the close anatomical relationship with the cranial nerves.

With the advance of diagnosis and the improvement in surgical techniques for vascular restoration and embolization, as well as the understanding of biological behavior of tumors, surgical treatment has developed and become safer.

## CASE REPORT

A 63-years-old white male patient, with a history of a painless mass on the left side of the neck during the preceding six months. He denied having dysphagia, hoarseness, buzzing, headache, arterial hypertension crises and tachycardia, or having similar cases among relatives.

On cervical examination, a motile, pulsatile mass, with no fremitus or murmur, was observed on the left side, next to the angle of the mandible.

**Figure 1:**
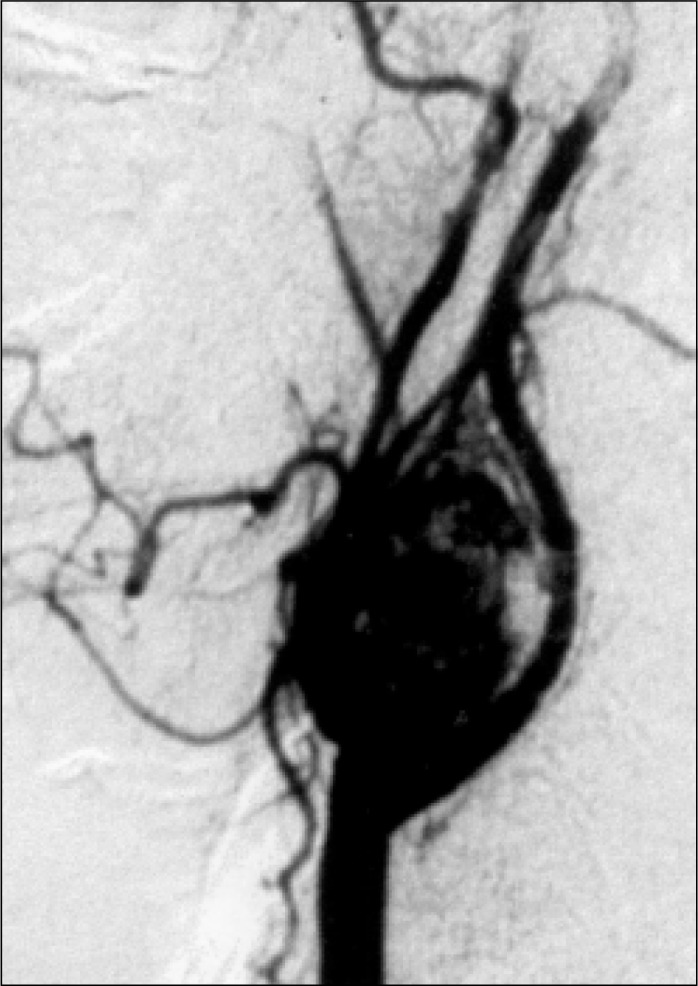
Arteriogram of typical carotid body paraganglioma (left side).

**Figure 2:**
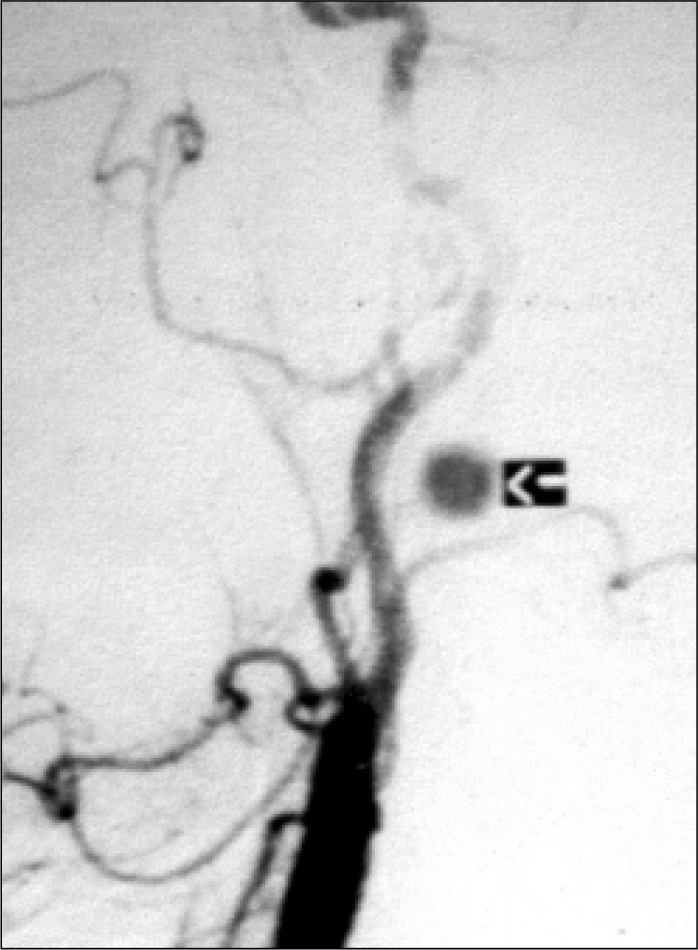
Arteriogram of the little carotid body paraganglioma (right side).

Computer tomography revealed a 5.5 x 3.8 x 3.2 cm mass on the bifurcation of the left common carotid artery. Arteriography demonstrated a hypervascularized mass occupying and deforming the left-side carotid bifurcation (Figure 1), as well as a small formation in the region of the right-side carotid bifurcation (Figure 2). The provisional diagnosis was bilateral carotid body paraganglioma.

Surgical treatment was preferred. On the left side, an oblique incision was made, following the anterior border of the sternocleidomastoid muscle, and extended proximally toward the mastoid process. For adequate exposure, the digastric and stylohyoid muscles were sectioned, and cranial isolation of the hypoglossal nerve was done. The vagus nerve with its pharyngeal and laryngeal rami was identified. The subadventitial dissection technique was employed and the external carotid artery was ligated, for better exposure of the tumor. There was no evidence of neurological deficit after the surgery.

Four months later the small mass on the right side was resected. Diagnosis was confirmed by the histological analysis of fragments of both masses.

## DISCUSSION

Carotid bodies are ellipsoid, red-brown formations, 5 to 7 mm in height by 2.5 to 4 mm in width, located in the posterior face of the common carotid artery bifurcation^1^ (as described by Von Haller in 1743).^2^ Reigner^3^ accomplished the first excision of a tumor at this location in 1880, but without the patient's survival. In 1886, Maydl^4^ was successful in the resection, but the patient became hemiplegic and aphasic. In 1889, Albert^5^ succeeded in removing the tumor.

The carotid trigone comprises the common carotid artery and its rami, the internal jugular vein and the vagus nerve. Among the structures that may be involved by these tumors and ought to be preserved during the surgical procedure are the hypoglossal nerve, the pharyngeal and superior laryngeal rami of the vagus nerve, the accessory nerve, the glossopharyngeal nerve, the mandibular ramus of the facial nerve and the cervical sympathetic chain.

Carotid body tumors are rare. Five hundred patients had been reported in the medical literature by 1972,^6^ six hundred by 1986^7^ and one thousand by 1988^8^ (a prevalence of 0.012% in autopsies).^9^

**Figure 3:**
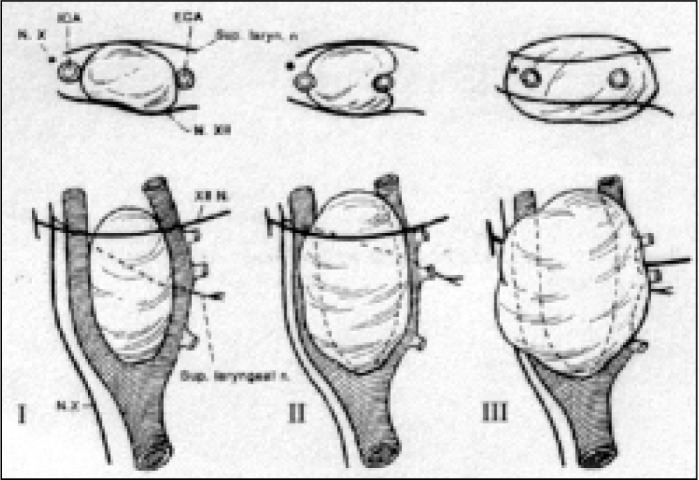
The classification of Shambin et al.^10^ of the difficulty of surgical resection.

Shamblin et al^10^ classified these tumors according to the degree of invasiveness of the arterial walls (Fig.3). Group I tumors are small and easily dissected in relation to the arterial wall; group II tumors are larger and adhered to the carotid adventitia, thus presenting difficulties in surgical resection; group III tumors are large and adhere intimately to the carotid adventitia (the highest complication rates are found within this group).^11^

There is no relationship between the histological appearance of the tumor and its behavior; that is, it is not possible to make a histological differentiation between slow growing tumors and rapid, invasive ones. The presence of metastases in the lymph nodes, or in more distant organs^12,13^ (1.7% to 50%)^12,14^, demonstrates the malignancy of these tumors.

Morbidity related to non-resected tumors is significant and includes paralysis of the cranial nerves, dysphagia, obstruction of the airways and invasion of the base of the skull.^12^

The sporadic form is the most common one and about 5% of the cases are bilateral.^9,15,16^ The second, rarer one, is the familial form, with a pattern of auto-somal, dominant inheritance, and about 32% of the cases are bilateral.^6^

Although asymptomatic in the initial stages, 93% of patients report the presence of a mass^14^ as the first manifestation of the disease. Other symptoms, such as accelerated growth of the mass (73%), headache or neck pain (35%), buzzing, dizziness, hoarseness (8%), dysphagia (8%) and syncope, occur in 75% of the patients. There is involvement of the cranial nerves, especially the vagus and the hypoglossal,^17^ in 10% of the cases. Likewise, the cervical sympathetic chain and the glossopharyngeal nerve may also be attacked.^14^

Differential diagnosis includes branchial cysts, carotid aneurysms, metastatic carcinomas, intravagal tumors, lymphomas and ectopic thyroid.^12^ On clinical examination these tumors are located below the angle of the mandible and posterior to the anterior border of the sternocleidomastoid muscle. They are motile in the lateral directions, but their relationship of proximity to the mandible limits their movements in the inferior-superior direction. The presence of fremitus and murmur in the region of the carotid bifurcation is not common. A mass in the pharyngeal region may be the most frequent indication (38%).^18^

Ultrasonography (color-Doppler) may confirm diagnostic suspicions of tumor presence. Computer tomography shows a mass at the carotid bifurcation and helps the analysis and detection of involvement of the cranial base, pharynx and jugular vein. Nuclear magnetic resonance supplies even greater details of information obtained from tomography and angioresonance provides evidence of tumor irrigation and atheroma plaque at the carotid bifurcation^19^.

Besides confirming the diagnosis (widening of the carotid bifurcation and hypervascularized mass; see Fig.1), arteriography detects the presence of occlusive carotid disease, the status of intracerebral blood circulation, tumor irrigation and the presence of small asymptomatic contralateral tumors, as well as allowing preoperative embolization.^10^ In this case reported here, it was fundamental in the diagnosis of the tumor on the right side.

Surgical treatment should be performed on the majority of patients. However, the slow growth of these tumors and poor surgical results in initial cases^10,20^ in relation to morbidity and mortality led many surgeons to believe that surgical treatment carried too many risks.

A significant decrease in death rates and vascular accidents^14^ can be attributed to the improvement in vascular restoration techniques, to subadventitial dissection of the tumor in relation to the arterial wall, broader incision for distal exposure of the internal carotid, optical magnification of the operative field, bipolar electrocauterization, multidisciplinary approaches, monitoring using electroencephalography during the operation, improvement in anesthetic procedures, introduction of heparin, early diagnosis and preoperative embolization.

Surgical results have improved in relation to the incidence of cerebrovascular accidents and mortality. Nevertheless, the high rate of cranial nerve dysfunction causes concern, having reached 50% in some cases^19^ (Table 1). The best results are reported from those cases where a multidisciplinary approach was used,^17^ with a vascular surgeon, head and neck surgeon, and preoperative embolization.^21^

**Table 1:** Perioperative complications of surgical resection for cervical paragangliomas

Authors	Time periods	N	Mortality (%)	Cranial nerve dysfunction (%)	Perioperative stroke (%)
Hallet Jr (Mayo Clinic) ^14^	1935 to 1965	70	6	46	23
	1966 to 1975	46	2	30	9
	1976 to 1987	37	0	40	2.7
Leonetti (Illinois)^19^	1988 to 1995	16	0	50	0

Restrictions on embolization include situations when there are tumors smaller than 3.0 cm or very small nutrient arteries, the duration of the examination (from four to six hours) and the likelihood of cerebral emboli.

The majority of authors have not managed to find confirmation of any benefits from radiotherapy for surgically untreatable patients.^10,12,15,18^

Meticulous surgical techniques, as well as knowledge of the anatomy of the region, are important issues for the decrease of the incidence of neurological lesions during carotid body tumor removal surgery. Arteriography may detect small masses, thus contributing to early diagnosis of these tumors.
